# Sensitivity Analysis of CLIMEX Parameters in Modeling Potential Distribution of *Phoenix dactylifera* L.

**DOI:** 10.1371/journal.pone.0094867

**Published:** 2014-04-10

**Authors:** Farzin Shabani, Lalit Kumar

**Affiliations:** Ecosystem Management, School of Environmental and Rural Science, University of New England, Armidale, New South Wales, Australia; Tennessee State University, United States of America

## Abstract

Using CLIMEX and the Taguchi Method, a process-based niche model was developed to estimate potential distributions of *Phoenix dactylifera* L. (date palm), an economically important crop in many counties. Development of the model was based on both its native and invasive distribution and validation was carried out in terms of its extensive distribution in Iran. To identify model parameters having greatest influence on distribution of date palm, a sensitivity analysis was carried out. Changes in suitability were established by mapping of regions where the estimated distribution changed with parameter alterations. This facilitated the assessment of certain areas in Iran where parameter modifications impacted the most, particularly in relation to suitable and highly suitable locations. Parameter sensitivities were also evaluated by the calculation of area changes within the suitable and highly suitable categories. The low temperature limit (DV2), high temperature limit (DV3), upper optimal temperature (SM2) and high soil moisture limit (SM3) had the greatest impact on sensitivity, while other parameters showed relatively less sensitivity or were insensitive to change. For an accurate fit in species distribution models, highly sensitive parameters require more extensive research and data collection methods. Results of this study demonstrate a more cost effective method for developing date palm distribution models, an integral element in species management, and may prove useful for streamlining requirements for data collection in potential distribution modeling for other species as well.

## Introduction

Species distribution models, ecological niche models (SDMs and ENMs) [1]–[3] and general bioclimatic models such BIOCLIM [4], are now acknowledged as essential tools in predicting a variety of future scenarios. Potential species distribution changes are one such application of these modeling tools [5]. Drawing on the distribution of the species and environmental data, a profile is compiled, relating distribution to variable environmental factors, a method termed the ‘environmental envelope approach' [6]. The founding principle on which this approach is based is that the primary determinant of range and potential range of plants and other poikilotherms is climate [7]. The environmental envelope falls within the parameters of the upper and lower tolerances of a species, which are used in the modeling process to create a habitat map that describes the environmental suitability of each location or potential location [6]. The environmental envelope approach has been the basis for the development of CLIMEX [8] and a number of other models [9], designed to model current or future distribution of a species [10], using data based on the environmental factors inherent within the natural distribution area of the species, to project levels of suitability for other previously uncolonized regions [11]. This modeling is valuable for the identification of potential localities where the species could be successfully introduced and survive, as well as for estimating the impact of a potential threat.

Despite widespread usage of these models, there are many challenges relating to inaccuracies of prediction [12] which may limit the usability of the output. One cause of inaccuracy in the modeling of species distribution relates to the inherent assumption that there is equilibrium between species and environment [6]. Such inaccuracies are at their most extreme in the modeling of distributions when a species has only recently been established in a particular locality. This becomes most pertinent where invasive species are not yet in equilibrium with the new environment and full distribution coverage is not yet fully established due to the particular dispersal rate of the species [13].

Further inaccuracies in output can be linked to data and calibration methods used for the establishment of parameters [12]. However sensitivity analysis provides a technique which may be used to better understand and thus eliminate the impacts of inaccuracy and error in output [14]. This type of analysis is invaluable for establishing which parameters have the greatest impact on the modeled results [15]. Species distribution software, such as CLIMEX, may show greater sensitivity in particular parameters than in others, which may impact on the projections themselves. Analysis of parameter sensitivity levels is vital for testing hypotheses regarding the impact of climate variables on distribution, and in addition assists in the understanding of which climatic factors cause the greatest impact on the populations of a particular species [16]. A global biome model's sensitivity to parameter value inaccuracies derived from literary sources was tested by Hallgren and Pitman [17], who showed that in most parameters the model demonstrated insensitivity but was sensitive in the case of photosynthesis-related parameters.

To help explain its relationships of climatic response, CLIMEX uses the documented geographical distribution of a particular species, from which it then projects potential climatic responses in other localities and under different scenarios of climate change. CLIMEX focuses on the distribution data of a species, in order to show more clearly the climatic conditions that support or restrict its growth [18]. It draws on various types of information to model a species' distribution potential, including current distribution, phenology and empirical observations of the effects of temperature and soil moisture on growth response. Reviewing climate-based software for estimating the potential distributions of species, Kriticos and Randall [9] rated CLIMEX as most suitable for performing weed risk assessments in that it supports fitting the model to global distribution of the plant, includes a mechanism to create climate change scenarios, and provides a view of the ecological response of the plant to climate. Another aspect favoring CLIMEX is that it indicates when factors other than climate are responsible for the limitation of geographical distribution, such as biotic interactions. It is essential that all parameters are biologically logical and that every possible record of positive locality has been included in the parameter-fitting exercise. Non-inclusion of localities of estimated suitability from the point of view of climatic conditions in the data indicates that other factors may be altering the distribution patterns used for positive data extrapolations for new regions, as is generally essential in the case of biotic invasions [18].

The current study has used CLIMEX in the development of a baseline model for the species *Phoenix dactylifera* L. (date palm), a crop of major economic value in Iran, Iraq, Egypt and Saudi Arabia, as well as in Spain and Turkey [19]–[24], the result of this modelling have been published in [10], [25]–[27]. Dates have been essential in supporting humans living in desert regions and thus the cultivation of the plant has had a major impact on Middle Eastern history. That dates are important is evidenced by great nutritional value of the plant (minerals, protein, vitamins, carbohydrates, fats, salts, and dietary fiber), its productivity and extensive yield life of up to 100 years [21], [28]–[35].

This study analyses parameter uncertainty and the effect thereof in quantifying date palm response to temperature, soil moisture and cold stress changes. Thus it identifies the parameters of functional importance to provide a greater understanding of the climatic factors that most impact on the plant's distribution. The Taguchi method [36] was utilized to provide the general framework for the recognition of parameter uncertainty and evaluation of its resultant effect on estimations and decisions. The study output provides an indication of those parameters requiring an accurate fit of the detailed data collection, as well as those that are relatively change insensitive and therefore requiring less investment into research and the collection of data.

## Materials and Methods

### Current Date Palm *(P. dactylifera)* Distribution in Iran

Satellite data were employed for the determination of date palm plantation sites in Iran. Data were collected from Landsat images with image resolution of 30 m [37], as well the Global Biodiversity Information Facility (GBIF) [38], Missouri Botanical Gardens' database (MBG) [39] and additional date palm literature resources in CAB Abstracts database [40]. The GBIF and MBG databases contain occurrence records of many plant species for the whole world. Further date palm literature supplementation was also utilized [10], [21], [29], [30], [41], [42]. A total of 145 records for *P. dactylifera* were obtained from the above sources, 19 of which had no geographical coordinates and were thus removed, leaving a working total of 126 records. To verify the point data accuracy, background satellite images were used to zoom in on each point for verification of the date palm presence in that locality. It should be mentioned that this section draws on a recent study that projected suitable regions for date palm cultivation in Iran under different climate change scenarios by 2030, 2050, 2070 and 2100 [25], [26].

### CLIMEX Software

The CLIMEX Version 3 software package [8], [42], [43] employs a model of eco-physiological growth with the inherent assumption that in a favorable season a population achieves a positive growth rate while an unfavorable season implies negative population growth [43]. Using the geographic range as reference, the parameters that describe response to climate of the species are inferred [44]. These parameters are in turn applied to the new climates to project the potential range of the species in alternative regions and different climate scenarios [45]. An index of annual growth (GI_A_) rates the population growth potential when climatic conditions are favorable. Conversely, the stress indices (cold, wet, hot or dry) indicate the survival probabilities in unfavorable conditions [43]. The annual growth index combines the temperature index (TI) and the moisture index (MI) representing the species' required levels of temperature and soil moisture for growth. Four parameters, minimum limit, optimum lower, optimum upper and maximum limit are applicable to the temperature and moisture indices, which are multiplied to produce the weekly growth index and the yearly average of this represents the annual growth index (GI_A_). The stress indices have two parameters, the threshold value and stress accumulation rate. The annual stress accumulation is exponential and once this value equals 1, the species will no longer be able to persist in that geographic region [43]. Growth and stress indices are calculated weekly and then integrated into the annual climatic suitability index, the ecoclimatic index (EI) on a scale between 0 and 100. A zero EI value denotes an unsuitable habitat where survival of the species is impossible; EI values between 1 and 10 denote marginal habitats; while values between 10 and 20 denote support for substantial populations and values greater than 20 denote high suitability [27], [44].

The methodology described in Sutherst and Maywald [8] was used to fit the growth and stress parameters. An in-depth account of these parameters is available in Sutherst and Maywald [8]. The Climate Research Unit (CRU), Norwich, UK, global meteorological dataset at 0.5° resolution [46] was supplied with the CLIMEX version used. This includes data from many locations worldwide, based on long-term monthly average maximum and minimum temperatures, rainfall, and relative humidity between the hours of 09:00 and 15:00, between 1961 and 1990. This meteorological dataset formed the basis for initial parameter-fitting.

### Model Framing

**Figure 1:**
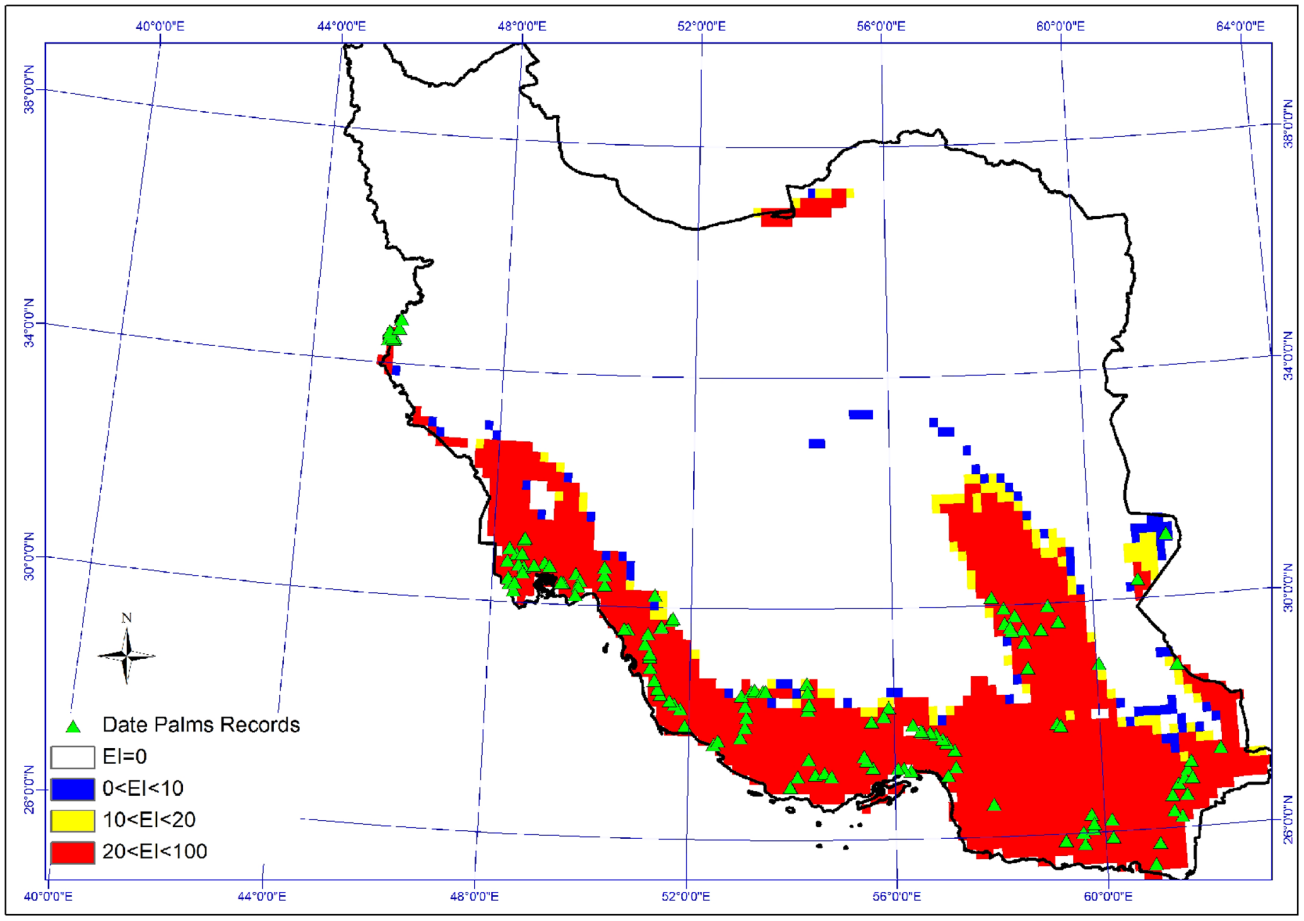
The current distribution of *P. dactylifera* L. and its potential distribution based on CLIMEX outputs at a country scale. (EI = 0, 0<EI<10, 10<EI<20 and 20<EI<100 means unsuitability, marginality, suitability and high suitability for date palm growth respectively).

CLIMEX model depicting climatic suitability for *P. dactylifera* was created using native and exotic distributions of date palm from a variety of data sources (Figure 1), [21], [23], [27], [29], [30], [47]–[56]. Table 1 summarizes all CLIMEX parameters. A detailed justification of these and their derivations is available in Shabani et al. [10].

**Table 1:** CLIMX parameter values used for *P. dactylifera* modeling.

Index	Parameter	Code	Values
	Limiting low temperature	DV0	14°C
**Temperature**	Lower optimal temperature	DV1	20°C
	Upper optimal temperature	DV2	39°C
	Limiting high temperature	DV3	46°C
	Limiting low soil moisture	SM0	0.007
**Moisture**	Lower optimal soil moisture	SM1	0.013
	Upper optimal soil moisture	SM2	0.81
	Limiting high soil moisture	SM3	0.9
**Cold Stress**	Cold stress temperature threshold	TTCS	4°C
	Cold stress temperature rate	THCS	−0.01/week
**Heat Stress**	Heat stress temperature threshold	TTHS	46°C
	Heat stress accumulation rate	THHS	0.9/week
**Wet Stress**	Wet stress threshold	SMWS	0.9
	Wet stress rate	HWS	0.022/week

### Taguchi Method

The Taguchi method applies orthogonal arrays to study many decision variables in relation to few experiments [57]. In other words, this statistical method efficiently decreases the number of iterations required in an optimization process [58], [59]. Compared to other optimization methods, including the Genetic Algorithm and the Particle Swarm Optimization, this method is easy to implement and can converge to the global optimum solution quickly [60]. Although the Taguchi method has been applied in many fields, including chemical and mechanical engineering [58], this method has rarely been used in climatic modeling studies. In the Taguchi method factors are divided into two main categories: controllable factors and noise factors [61]. Noise factors include those over which the experimenter cannot exert direct or exact control. Since noise factor elimination is impractical and sometimes even impossible, the Taguchi method aims to minimize noise effects and to calculate optimal levels of vital controllable factors, incorporating the concept of robustness [62]. Taguchi also proportions the significance of specific factors in terms of effects on the objective function [36]. Taguchi also proposes transformation of reiteration data to another value as a variation measure [63]. This transformation is based on the signal-to-noise (SN) ratio and explains why a particular parameter design is described as robust [62]. The term ‘signal’ represents the desired values (mean response variables), while ‘noise’ represents the undesired values (standard deviations), with the aim of maximizing the signal-to-noise ratio. Furthermore, objective functions are classed by Taguchi into three groups: 1 smaller the- better; 2 larger-the-better; and 3 nominal-best. A major feature of Taguchi method is the generation of sensitivity results within a single scale for different parameters, enabling users to compare sensitivity of specified parameters.

We established 14 Taguchi method factors, the same as the number of CLIMEX parameters and chose 54 runs, which is the maximum number of iterations and gives maximum accuracy, with 2×1 and 3×25 Array in a mixed level design to optimize sensitivity analysis accuracy.

A Taguchi-based sensitivity analysis was performed to quantify *P. dactylifera* L. response to temperature, soil moisture and cold, wet and heat stress parameter changes. Here the parameter values were based on Table 2, and in a few cases where the values fell outside the CLIMEX range, the values were adjusted. Adjusted models were then re-run after each parameter value change and thereafter the area of suitable and highly suitable categories were calculated for the baseline and for the final adjusted models, to assess the different parameters' sensitivity levels. In summary, 12 out of 54 different sets of control factors derived by the Taguchi method for potential distribution of *P. dactylifera* L. based on CLIMEX parameters is summarized in Table 3.

**Table 2:** Factors and levels of potential distribution of *P. dactylifera* L. based on CLIMEX.

	Level 1	Level 2	Level 3
**SM0**	0.005	0.007	0.01
**SM1**	0.011	0.013	0.017
**SM2**	0.5	0.81	1
**SM3**	0.6	0.9	1
**DV0**	10	14	18
**DV1**	15	20	26
**DV2**	30	39	45
**DV3**	40	46	50
**TTCS**	2	4	5
**THCS**	−0.05	−0.01	0
**TTHS**	40	46	50
**THHS**	0.7	0.9	1
**SMWS**	0.6	0.9	1
**HWS**	0.018	0.022	0.029

**Table 3:** A sample set of control factors derived by Taguchi method for potential distribution of *P. dactylifera* L. based on CLIMEX parameters.

							Parameters								
Sample Runs	DV0	DV1	DV2	DV3	SM0	SM1	SM2	SM3	TTCS	THCS	TTHS	SMWS	HWS	THHS	Areas of suitability (km^2^)
1	1	1	1	1	1	1	1	1	1	1	1	1	1	1	373977
2	1	1	2	2	2	2	2	2	1	1	1	1	1	1	1435284
3	1	2	2	2	3	3	1	1	1	1	2	2	3	3	1190953
4	1	2	2	2	3	3	1	1	2	2	3	3	1	1	427252
5	1	3	1	2	1	3	2	3	3	1	3	2	1	2	1345530
6	1	3	2	3	2	1	3	1	2	3	2	1	3	1	323326
7	1	3	2	3	2	1	3	1	3	1	3	2	1	2	1364426
8	1	3	3	1	3	2	1	2	3	1	3	2	1	2	1235830
9	2	2	2	3	1	2	1	3	3	1	2	3	2	1	1176781
10	2	2	3	1	2	3	2	1	2	3	1	2	1	3	539052
11	2	3	1	3	2	3	1	2	2	1	3	1	2	3	1045823
12	2	3	3	2	1	2	3	1	1	3	2	3	1	2	315978

Incremental model EI values from the sensitivity analysis were plotted and compared with those of the baseline model. Where an altered incremental model parameter was found to be highly sensitive, a large change in EI value was expected. However where a parameter was not highly sensitive, we expected the baseline and incremental model EI values to be similar. Suitability changes were also assessed by area mapping, where suitability levels changed in terms of the suitable or highly suitable categories, for high level sensitivity parameters. All validation data changes related to parameter changes were further assessed by noting all changes in the number of occurrence records falling within each of the suitability categories.

## Results

### Historical Climate

A comparison of the model for climate suitability with the date palm distribution for Iran shows consistency in the correlation of the modeled EI with current distribution of *P. dactylifera*. Suitable climatic conditions for *P. dactylifera* lie between 48° E and 52° E, 57° E and 60° E, with large areas between 25° N and 29° N in central Iran (Figure 1).

**Figure 2:**
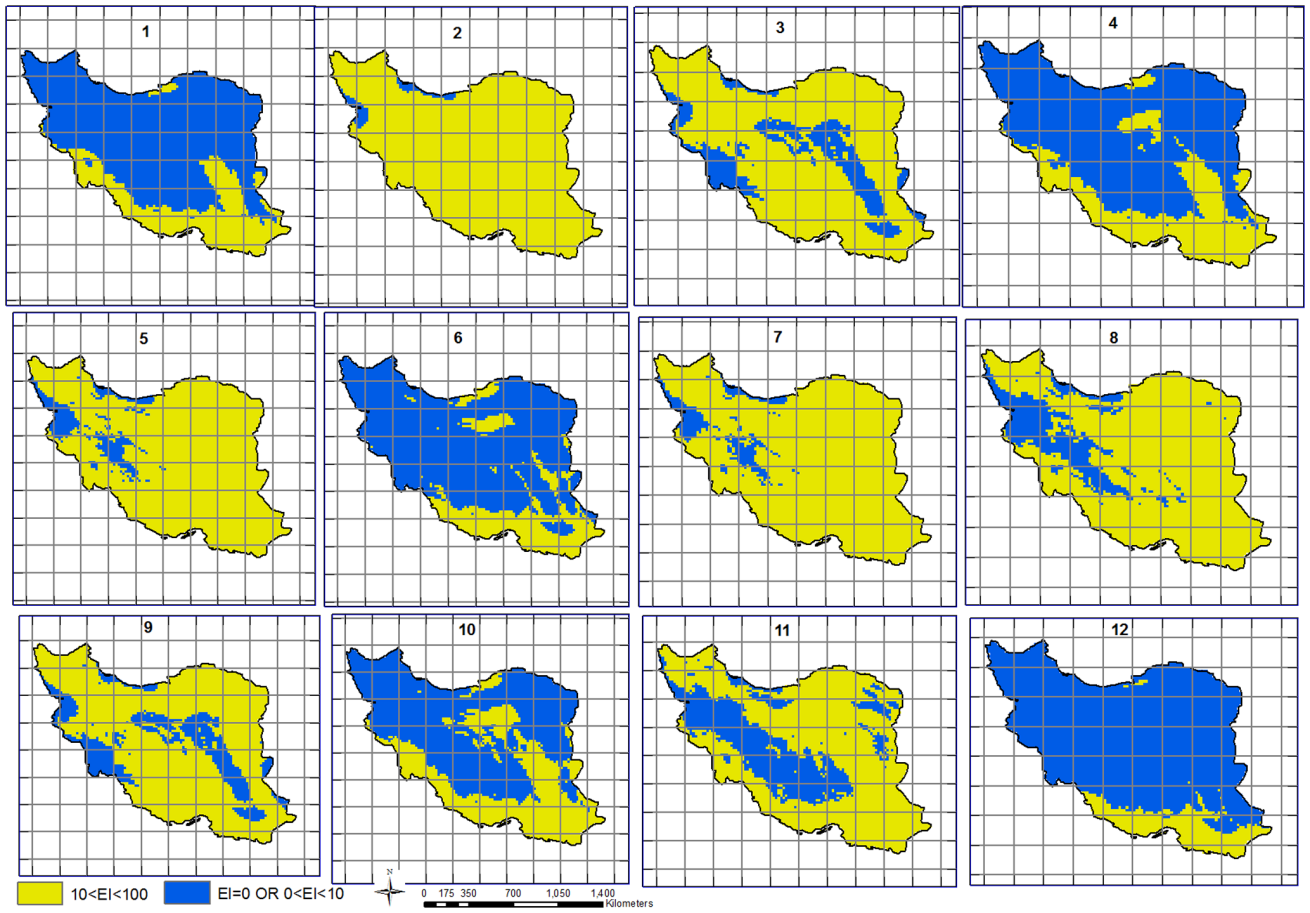
12 sample results out of 54 different control factors based on Taguchi method for *P. dactylifera* L. Area changes in suitable and highly suitable categories when sensitivity analysis was taken. The values for the various parameters used are those given in table 3.

**Figure 3:**
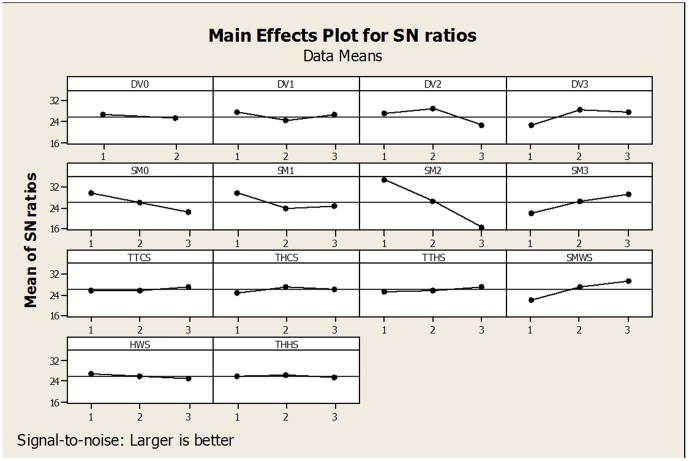
Sensitivity analysis of the 14 parameters in CLIMEX for *P. dactylifera* L.

A sample set of temperature parameter changes from the baseline model and the resultant impact on distribution are shown in Figure 2, while the final sensitivity analysis results, using the adjusted values of the Taguchi method, are illustrated in Figure 3. In the Taguchi method, the flat sensitivity lines indicate that that particular variable is not sensitive.

The potential distribution of *P. dactylifera* L. baseline model was highly sensitive to change in DV2 (low temperature limit) and DV3 (high temperature limit). In other words, changing DV2 and DV3 values produced markedly increased or decreased suitable and highly suitable areas. These changes produced a northward shift in the distribution of the species, creating larger areas in central and northern Iran. Western Iran showed a similar trend, in areas that were originally unsuitable or marginally suitable for growth of date palm. Comparing sensitivity also showed that in CLIMEX, DV1 was more sensitive to change than DV0, but not as much as DV2 and DV3 (Figure 3). In other words, suitable and highly suitable areas changed less rapidly with DV1 adjustment from the baseline. Figure 3 also demonstrates that the values chosen for the baseline model DV2 and DV3 parameter were accurate, since the SN ratio mean was optimum.

In regard to the four soil moisture parameters, the upper optimal soil moisture level (SM2) proved most sensitive to changes (Figure 3). A small increase or decrease in this parameter projected large areas that could become suitable or highly suitable in the modeled suitability. SM0, SM1 and SM3 also proved sensitive to changes (Figure 3). Figure 3 also shows that level one in the parameters of SM0, SM1 and SM2 produced the highest SN ratio mean, while conversely the optimum mean of SN ratios for SM3 was observed at level three (Figure 3). In other words, SM0, SM1, SM2, and SM3 changes produced markedly changes in date palm distribution.

The cold stress temperature threshold (TTCS), cold stress temperature rate (THCS), heat stress accumulation rate (THHS) heat stress temperature threshold (TTHS) and the wet stress rate (HWS) demonstrated insensitivity to change. The alteration in the wet stress threshold (SMWS) thus conversely reflects the changes in EI when this parameter is modified (Figure 3).

## Discussion

This study delineates the relationship in Iran between climate and *P. dactylifera* L. distribution. CLIMEX and the sensitivity analysis based on the Taguchi method illustrated informatively specific parameters that had the most effect on the modeled distribution of *P. dactylifera* L. Results demonstrate that distribution of *P. dactylifera* L. is highly sensitive to DV3 and DV2 (the limiting high and upper optimal temperature) changes as well as in SM3 and SM2 (the limiting high soil moisture and the upper optimal moisture) parameters (Figure 3). Additionally, date palm distribution was slightly sensitive to SM1 and SM0 (lower optimal soil moisture and lower limit soil moisture) and SMWS (wet stress threshold) parameters. These results contrasted with the results of the sensitivity analysis for *Lantana camara* L. [64], rated as one of the ten most destructive weeds in the world [65]. Taylor [64] showed that the distribution of lantana was highly sensitive to DV0, DV3 and SM0 parameter changes. Additionally, Taylor [64] showed that DV0 and DV3 changes had the substantially modified suitable and highly suitable lantana location in Australia. The total difference in climatic parameter requirements for these two species is possibly responsible for such markedly differences. For example, if the soil is not waterlogged for prolonged periods, lantana is tolerant of up to 3000 mm of rainfall annually [66], [67] while documentation shows that 78.74 mm of rainfall over an 8-day period caused over 50% loss in yields of date palm, while 86.36 mm over 10 days resulted in a 15% loss on date palm farms in some countries [10]. Another difference in essential climatic parameters between lantana and date palm is upper optimal temperature, being 39°C and 30°C respectively [11], [64].

Generally, where an area indicated decreased suitability, this was reflected in a decrease in the number of highly suitable category records, while records increased in the unsuitable and marginal categories. In certain cases, there was an increase of records in the suitable category, suggesting that some highly suitable areas were rendered just suitable by adjustment of parameters. Conversely, an increased suitable area generally reflected in increased records in the highly suitable and suitable categories and decreased records in the unsuitable and marginal categories. Here, a decrease in records in the suitable category suggests that certain suitable areas became highly suitable with changes in parameter value.

The SM3, SM2 and SM0 moisture parameters were the most sensitive and showed greater shifts in distribution when altered, compared to SM1. A high level of sensitivity of this species within these parameters was demonstrated by these results. The cold stress temperature threshold (TTCS) and cold stress temperature rate (THCS) also demonstrated minimal levels of sensitivity to change. Acute specific changes in the sensitivity analysis to parameter values were facilitated by an in-depth understanding of the relationship of the species with climatic factors, based on the extensive date palm distribution in Iran and the relevant available research data. Table 3 gives the changes in area of suitability as the parameters are changed. Sample runs 2, 5, 7 and 8 (See Table 3 for detailed parameter settings) produced the highest suitable areas, while sample runs 1, 6 and 12 had least area.

Distribution predictive modeling is a useful tool for control and management of a species. Such models provide potential distribution mapping of a species, allowing policy makers at the national and international level to make well-informed decisions regarding management of their market. Predictive modeling techniques, such as CLIMEX, derive data on climatic requirements of a target species from species' geographic distribution data, which supports parameter-fitting in the development of the model. However, a particular species of interest may have greater sensitivity to certain climatic factors than to others, and these varying sensitivity levels may complicate distribution predictive modeling. Those parameters highly sensitive to change will have a greater impact on the output of the model than the relatively insensitive parameters. Sensitivity analysis highlights parameters of greater or lesser relative importance, useful for improving data collection [68]. It is for good reason that formal sensitivity analyses are advocated as the most effective method for the evaluation and refining of improvements to model input data [69].

For the continuation of model output development and the collecting of data for fitting of highly sensitive parameters with greater accuracy, further research is essential. Conversely, the cost effectiveness of the collection of additional data for relatively insensitive parameters may not be valid, if the model output improvements are minimal. However research towards the quantification of parameter sensitivity and the resultant refinement of model outputs are useful as a means to improving confidence in parameter estimates [70], leading to the most cost effective management strategies. Toward this end, perhaps this study's most encouraging finding is that eight of the fourteen parameters tested impacted strongly on potential date palm distribution, namely the DV1, DV2, DV3 and SM0, SM1, SM2, SM3 and SMWS parameters. Thus, where resources and support are limited, it would be most expedient to research and incorporate a wider range of alternative data sources towards fitting these eight parameters with increased accuracy.

The sensitivity analysis results also signal the need for caution regarding regional and global models of climate change in projecting date palm distributions, that all models and climate change scenarios in the future include ranges for both temperature and rainfall variation. The Special Report on Emissions Scenarios (SRES) A1F1 fossil intensive scenario projects an increase in global average temperatures of 2.4 to 6.4°C, making this of vital interest [71]. The lower end of this spectrum projects an increase of 1.1 to 2.9°C based on the SRES B1 scenario. This projected increase in the average global temperature would have drastic implications for date palm, in terms of the level of sensitivity its distribution has demonstrated regarding changes to the upper and lower temperature tolerance limits (DV2 and DV1).

CLIMEX's central assumption is that the primary determinant of the geographical distribution of a species is climate. Thus dispersal potential, biotic interactions, soil type, land-use and disturbance activities and other non-climatic factors are not explicitly incorporated in the modeling process. These factors can however be incorporated after the climate modeling process has been executed [26]. Additionally the inclusion of native as well as exotic distribution data should include any effects from the release of natural enemies [72] apparent in the exotic range of species such as date palm. This more clearly defines its fundamental niche [73]. Thus, the methodologies that refine the data necessary for date palm potential distribution modeling tools are invaluable. We used a sensitivity analysis for the identification of the CLIMEX parameters and consequent climate aspects that most influenced date palm potential distribution in Iran. This approach is extremely useful in the streamlining of the requirements for data collection for the modeling of potential distribution.
